# Nonlinear Probability Weighting in Depression and Anxiety: Insights From Healthy Young Adults

**DOI:** 10.3389/fpsyt.2022.810867

**Published:** 2022-03-24

**Authors:** Kosuke Hagiwara, Yasuhiro Mochizuki, Chong Chen, Huijie Lei, Masako Hirotsu, Toshio Matsubara, Shin Nakagawa

**Affiliations:** ^1^Division of Neuropsychiatry, Department of Neuroscience, Yamaguchi University Graduate School of Medicine, Ube, Japan; ^2^RIKEN Center for Brain Science, Wako, Japan

**Keywords:** decision-making, reward, risk preference, risk aversion, probability weighting, depression, anxiety, computational psychiatry

## Abstract

Both depressive and anxiety disorders have been associated with excessive risk avoidant behaviors, which are considered an important contributor to the maintenance and recurrence of these disorders. However, given the high comorbidity between the two disorders, their independent association with risk preference remains unclear. Furthermore, due to the involvement of multiple cognitive computational factors in the decision-making tasks employed so far, the precise underlying mechanisms of risk preference are unknown. In the present study, we set out to investigate the common versus unique cognitive computational mechanisms of risk preference in depression and anxiety using a reward-based decision-making task and computational modeling based on economic theories. Specifically, in model-based analysis, we decomposed risk preference into utility sensitivity (a power function) and probability weighting (the one-parameter Prelec weighting function). Multiple linear regression incorporating depression (BDI-II) and anxiety (STAI state anxiety) simultaneously indicated that only depression was associated with one such risk preference parameter, probability weighting. As the symptoms of depression increased, subjects’ tendency to overweight small probabilities and underweight large probabilities decreased. Neither depression nor anxiety was associated with utility sensitivity. These associations remained even after controlling covariates or excluding anxiety-relevant items from the depression scale. To our knowledge, this is the first study to assess risk preference due to a concave utility function and nonlinear probability weighting separately for depression and anxiety using computational modeling. Our results provide a mechanistic account of risk avoidance and may improve our understanding of decision-making deficits in depression and anxiety.

## Introduction

Depressive and anxiety disorders (hereafter, depression and anxiety) are two most prevalent and disabling mental disorders that greatly limit people’s daily activities (1, 2). One frequently reported deficit common to both disorders is impaired decision-making under risk or risk preference. For instance, both depression (3–5) and anxiety (6–10) have been associated with enhanced risk avoidant behaviors. Risk neutrality is typically considered optimal behavior while excessive risk avoidance may reduce the opportunities of potentially rewarding stimuli and lead to suboptimal outcomes. In fact, these behaviors have been considered an important contributor to the maintenance and recurrence of depression and anxiety, and have been employed a primary treatment target [for reviews, Jacobson *et al.* (11) and Pittig *et al.* (12)].

Despite these fruitful findings, two fundamental questions remain to be addressed. Firstly, given the high comorbidity between depression and anxiety (13), their independent association with risk preference remains unclear. Are they both, or is just one of them, associated with changed risk preference? Secondly, due to different definitions of “risk” in psychology and neuroeconomics and the involvement of multiple cognitive computational factors in decision-making processes, the precise underlying mechanisms of risk preference in depression and anxiety are unknown. Whereas risk commonly refers to the probability of a choice leading to an aversive outcome such as a loss or harm in psychology, it is defined as the variability of possible outcomes of a choice in behavioral economics and neuroeconomics (14).

Notably, previous studies of risk preference in depression and anxiety have generally employed the psychological definition of risk. For instance, two mostly widely employed tasks for the evaluation of risk preference in depression and anxiety are the Iowa Gambling Task (IGT) and the Balloon Analog Risk Task (BART). In the IGT (15), subjects are asked to choose one of four decks of cards to maximize their reward. Unknown to them, two of the four decks are disadvantageous such that although they bring a higher reward, occasionally they are associated with a much higher penalty and frequent choice from these decks will result in long-term loss. The other two decks are advantageous and although they bring lower gains, the occasional penalties are also lower. The proportion of choices taken from the advantageous decks is typically used as an index of task performance and risk-aversion. In the BART (16), subjects are asked to pump a balloon to earn money. Each pump expands the balloon and earns a fixed amount of money. Each pump, however, also increases the chance of the balloon exploding, which causes the loss of all money earned from that balloon (as one trial). The average number of pumps on unexploded balloons is used as an index of risk-taking propensity. Based on these tasks, it has been suggested that patients with depression and anxiety and people with high symptoms of depression and anxiety show reduced risk-taking or increased risk-aversion (3, 5, 7, 8).

However, it has to be noted that these tasks do not allow pure, reliable evaluation of risk preference, because in addition to risk preference (according to the economic definition), both tasks also involve the decision-making process of reinforcement learning (of each card-outcome contingency and balloon exploding probability) and loss aversion (toward penalty and balloon exploding which causes the loss of all reward). These three are different cognitive computational processes and rely on distinct neurobiological mechanisms (14, 17, 18). This may explain why previous studies with the IGT have generated conflicting results, for instance, both impaired and enhanced performance have been reported in individuals with high depression [Must et al. (19), Cella et al. (20) versus Smoski et al. (3)] and high anxiety [Miu et al. (21) versus Mueller et al. (8)]. To clarify whether depression and anxiety are associated with risk preference, one has to use more appropriately designed tasks and computational modeling of the decision-making process that allow the differentiation of relevant cognitive computational processes.

**FIGURE 1:**
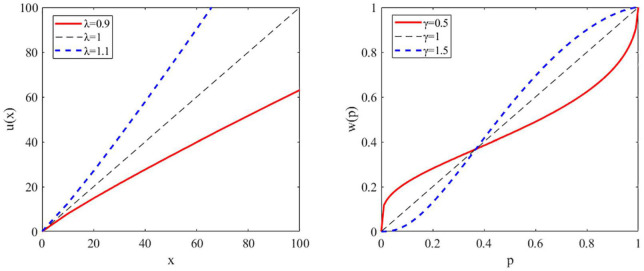
Illustration of the utility and probability weighting function. Left panel: a representative plot of the utility function for risk-averse, risk neutral, and risk-seeking individuals. Utility (u) as a function of reward amount or magnitude (x). Right panel: a representative plot of the probability weighting function showing the decision weight (w) as a function of objective probability (p).

In economics, risk is defined as outcome variance and risk aversion refers to the preference of outcomes with high certainty. For instance, an individual preferring an option with a sure outcome (e.g., $50 guaranteed) over another with an unsure outcome that has equal or greater expected value (e.g., 50% chance of getting $100) is said to be risk-averse, while an individual with the opposite preference is said to be risk-seeking. This risk preference is captured by a utility or utility sensitivity function (e.g., a power function), for which linear utility functions indicate risk-neutrality, concave utility functions indicate risk aversion, and convex functions indicate risk seeking (see Figure 1, left panel). To account for mixed risk preference at small versus large probabilities, Prospect theory (22, 23) introduced the probability weighting function and suggested that most people tend to overweight small probabilities (i.e., risk seeking in the case of gain) and underweight large probabilities (i.e., risk aversion in the case of gain). Here, a linear function indicates the subjective probability equals the objective probability indicates risk-neutrality, and a nonlinear function indicates that people have different risk preference at small versus large probabilities (see Figure 1, right panel). Therefore, the combination of a utility function and a probability weighting function can more thoroughly capture people’s risk preference.

In the present study, we set out to investigate the common versus unique cognitive computational mechanisms of risk preference in depression and anxiety using a reward-based learning-free decision-making task and computational modeling based on the above economic theories. Specifically, we attempted to dissect risk preference into two parameters, utility sensitivity and probability weighting, and investigate their independent association with depression and anxiety using multiple linear regression analysis.

## Materials and Methods

### Participants

This research was part of an ongoing cohort study conducted to predict the mental health of young adults. The study was approved by the Institutional Review Board of Yamaguchi University Hospital and performed according to the latest version of the Declaration of Helsinki. The inclusion criteria were being 20–39 years old. The exclusion criteria were (1) having any self-reported psychiatric disorders, (2) receiving medical examinations due to suspicion of any psychiatric disorders, (3) being suspected of psychiatric disorders and diagnosed as having any psychiatric disorders by a psychiatrist, or (4) being unable to perform the tasks or questionnaires for this study.

Data collected at the baseline of the study during the year 2019 were used for the data analysis here. Specifically, 68 participants agreed to participate in this study and provided written informed consent. No participant met any of the exclusion criteria. We considered this sample size appropriate for our analysis here, because based on a priori power analysis conducted with G*Power version 3.1.9.7 (24), to detect a significant regression coefficient of moderate effect size [*f*^2^ = 0.15; (25)] with alpha = 0.05, power = 0.8, and two predictors (i.e., depression and anxiety), 43 participants are required.

### Demographic Information

Participants first filled out questionnaires about their demographic characteristics, including gender, age, occupation, education level, and socioeconomic status such as whether they had made a student loan as an undergraduate student, their parents’ education levels and family income.

### Decision-Making Task

**FIGURE 2:**
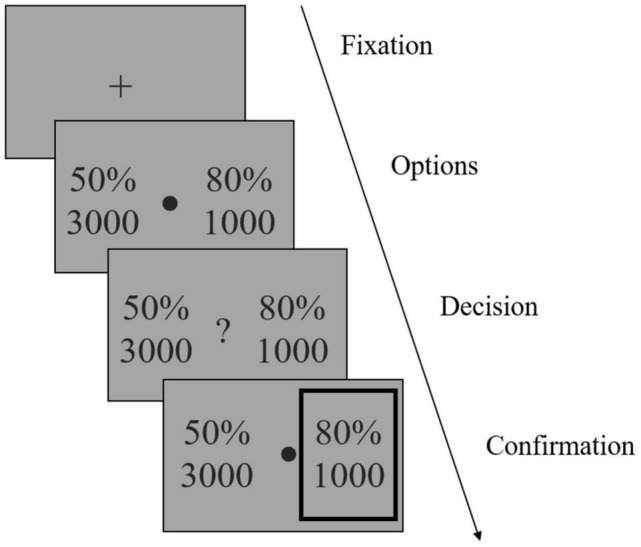
Illustration of the reward-based decision-making task. After a fixation phase, two gambling options were shown (Options phase). Each option consisted of a reward magnitude (in JPY) and the probability of receiving that magnitude of reward. Participants were instructed to choose one option to maximize their reward, by pressing one of two predefined arrow keys once a question mark occurred (Decision phase). The chosen option was then highlighted by a gray frame (Confirmation phase). Note that the gambling options shown in the figure were not actual stimuli used in the study.

We adapted an established task design (26). The task was programmed with MATLAB R2018b (MathWorks) and Psychtoolbox 3.^1^ The task had 120 trials and was conducted in three sessions, each separated by a short break. In each trial (Figure 2), participants were instructed to choose between two gambling options to maximize their reward. Each option consisted of a reward magnitude (in JPY, the lower number) and the probability of receiving that magnitude of reward (the upper number). For the option pairs of reward magnitude and probability, we used the stimuli generated by Hsu et al. (26) (Supplementary Table 1), but replaced the original amount in dollar with that in JPY by multiplying 100 (as an approxiate of the exchange rate). Furthermore, we presented the probabilities as percentages instead of the orignal ratios (e.g, 40/100) used by Hsu et al. (26) to faciliate perception. To ensure that participants were focusing on the task, after a randomly selected trial in every 15 trials, we inserted a test trial (eight in total) which had a correct answer (e.g., 30%, 5,000 versus 50%, 5,000).

After a fixation phase (or inter-trial interval) of 1.5 s, the stimuli were shown on the screen for 3 s (Options phase), after which a question mark occurred and participants indicated their choice by pressing one of two arrow keys within 3 s and as soon as possible once they decided which to choose (Decision phase). The chosen option was then highlighted by a gray frame (Confirmation phase). Participants were informed that failing to respond within the decision phase would be counted as no response and lead to no reward on that trial.

Each subject received a fixed payment of about 3,000 JPY for participating in the study. We did not implement the performance-adjusted payment because the final aim of our study was to develop useful tools for predicting mental health problems in a public health setting. For such purpose, it is impossible to pay people a certain amount of money based on their task performance in any public health screening tools. Furthermore, previous studies have shown that people’s decision-making with hypothetic rewards highly resembles that with real rewards (27).

### Depression and Anxiety

The Beck Depression Inventory-II (BDI-II) and the state anxiety subscale of State-Trait Anxiety Inventory (STAI-Y1) were employed to evaluate the symptoms of depression and anxiety, respectively. Since BDI-II also includes two primary symptoms of anxiety, namely agitation and irritability, we also created another variable of depression BDI-pure by excluding these two items.

### Data Analysis

IBM SPSS Statistics 26 (IBM Corp., Armonk, NY, Untied States) and MATLAB R2018b (The MathWorks, Inc., Natick, MA, United States) were used for data analysis. All statistical tests were two-sided and *p* < 0.05 was considered significant.

For the computational model-based analysis of the behavioral data, we fitted six models to participants’ choice (Table 1). Two models used a value function that considered only reward magnitude or probability (i.e., Models 1 and 2). Four models (i.e., Models 3–6) used a value function that considered both magnitude and probability with or without a utility function (i.e., a power function) and/or probability weighting function (i.e., the one-parameter Prelec weighting function).

**TABLE 1:** Model specification and fitting results.

Model No.	Model description	Equation	Free parameters	AIC
1	Magnitude only	*V*(*X*) = *r*	β	165.57
2	Probability only	*V*(*X*) = *p*	β	113.82
3	Magnitude and probability	*V*(*X*) = *rp*	β	161.38
4	Magnitude with utility function and probability	*V*(*X*) = *r*^λ^*p*	λ, β	105.47
5	Magnitude and probability with probability weighting	*V*(*X*) = *re*^−(−*log*⁡*p*)^γ^^	γ, β	157.85
6	**Magnitude with utility function and probability with probability weighting**	*V*(*X*) = *r*^λ^*e*^−(−*logp*)^γ^^	**λ, γ, β**	**96.03**

*The winning model is shown in bold.*

For the utility function parameter λ, 1 indicates risk-neutrality, <1 indicates risk aversion, and >1 indicates risk seeking. For the probability weighting function parameter γ, 1 indicates rational probability weighting, <1 indicates overweighting of small probabilities and underweighting of large probabilities, and >1 indicates the opposite. Participants were then assumed to choose actions stochastically according to a sigmoidal probability distribution, with an inverse temperature parameter β adjusting the degree of stochasticity in participants’ choices. Following Hsu et al. (26), the models were fitted to each participant’s behavior using maximum likelihood estimation. The estimation was conducted using the *fmincon* command of MATLAB 2018b. Model selection was based on Akaike information criterion (AIC), which puts a penalty on the increasing number of free parameters. The estimated parameters of the winning model were then used for subsequent data analysis. To investigate the independent association between depression, anxiety, and risk parameters, we conducted multiple linear regression, with the risk parameters as dependent variables and depression and anxiety as independent variables. We also included demographic or socioeconomic factors as covariates for the regression analysis if they were correlated with depression, anxiety, or any of the risk parameters. There were two missing values with the data of father education level, which were replaced with multiple imputation (imputed five times by ordinal logistic regression with other demographic factors as predictors). Father education and mother education were coded as 1 for elementary school level, 2 for junior high school level, 3 for senior high school level, 4 for vocational school level, 5 for undergraduate level, 6 for master’s level, and 7 for doctorate level. The data of family income had ten missing values and therefore the variable was excluded from data analysis. The normal P–P plot of regression standardized residual of each regression model was confirmed and presented in Supplementary Figure 1 (for regression reported in Table 2) and Supplementary Figure 2 (for regression reported in Table 3). We did not detect any obvious multicollinearity (i.e., variance inflation factors all <1.7) or homoscedasticity issue with the regression models. Given our small set of predictors and that we were not clear which was the best predictor, we used the standard “Enter” method for the regression models.

**TABLE 2:** Results of the multiple linear regression using BDI.

	Independent variables	Dependent variable: γ			Dependent variable: λ		
		Unstandardized *B* (95% CI)	Standardized beta	*p*	Unstandardized *B* (95% CI)	Standardized beta	*p*
Model 1	BDI	**0.015 (0.002, 0.029)**	**0.389**	0.027*	−0.007 (−0.019, 0.005)	−0.197	0.268
	STAI-Y1	−0.004 (−0.016, 0.008)	−0.118	0.494	0.009 (−0.001, 0.019)	0.303	0.091
Model 2	BDI	**0.014 (0.000, 0.027)**	**0.346**	0.043*	−0.006 (−0.018,0.006)	−0.185	0.305
	STAI-Y1	−0.002 (−0.014, 0.009)	−0.071	0.674	0.008 (−0.002,0.019)	0.290	0.111
	Mother education	**0.115 (0.006, 0.224)**	**0.277**	0.039*	−0.027 (−0.127,0.074)	−0.075	0.594

*p < 0.05. Significant results are shown in bold.

**TABLE 3:** Results of the multiple linear regression using BDI-pure.

	Independent variables	Dependent variable: γ			Dependent variable: λ		
		Unstandardized *B* (95% CI)	Standardized beta	*p*	Unstandardized *B* (95% CI)	Standardized beta	*p*
Model 3	BDI-pure	**0.017 (0.003, 0.032)**	**0.407**	0.018*	−0.006 (−0.019, 0.006)	−0.175	0.317
	STAI-Y1	−0.004 (−0.015, 0.007)	−0.120	0.474	0.008 (−0.002, 0.018)	0.285	0.105
Model 4	BDI-pure	**0.016 (0.002, 0.030)**	**0.363**	0.030*	−0.006 (−0.019, 0.007)	−0.163	0.358
	STAI-Y1	−0.002 (−0.014, 0.009)	−0.074	0.652	0.008 (−0.002, 0.018)	0.273	0.127
	Mother education	**0.113 (0.005, 0.222)**	**0.273**	0.040*	−0.027 (−0.128, 0.074)	−0.076	0.590

*p < 0.05. Significant results are shown in bold.

### Additional Analysis

Given the considerable correlation between BDI and STAI-Y1, we also took two additional approaches to investigating their independent association with the risk parameters. Firstly, we conducted a principle component analysis (PCA, with Varimax rotation) with the individual items of BDI and STAI-Y1 and extracted seven factors that account for 64% of the total variance. The scree plot is shown in Supplementary Figure 3 and the item structures of the seven factors are shown in Supplementary Table 1. As can be seen, Factors 1 and 7 primarily measure anxiety and Factors 3–6 primarily measure depression, while Factor 2 captures both anxiety and depression. For the calculation of the factor score, we employed two methods, one was a commonly used non-refined method, namely simply summing the raw scores of all items loading on the factor, the other was a refined method known as the Anderson-Rubin score (28). The correlation between the score of the extracted factors and the risk parameters is shown in Supplementary Table 2. In brief, a consistent correlation was identified between Factor 3 (which primarily measures depression) and γ across the factor scores. In contrast, the correlation between Factors 5 (which primarily measures depression) and λ was significant for the sum of raw score but not the Anderson-Rubin score. Unfortunately, these correlations became nonsignificant after partialling out mother education.

Secondly, we examined the correlation between individual items of BDI and STAI-Y1 and selected items having the fewest correlations with items from the other scale (Supplementary Table 3). For depression, eight items were selected (i.e., symptoms of sadness, suicidal thoughts, loss of interest, worthlessness, changes in sleep, appetite, fatigue, and loss of interest in sex). For anxiety, six items were selected (i.e., feelings of calm, secure, tense, strained, at ease, and confused). Although the selection was rather arbitrary, we considered these symptoms representative of depression and anxiety, respectively. We then ran correlation analysis between the total scores of the selected items (hereafter Depression-selected and Anxiety-selected) and the risk parameters (Supplementary Table 4). It was found that Depression-selected but not Anxiety-selected was associated with γ; neither was associated with λ. These results remained even after partialling out mother education. The scatter plot of the associations is shown in Supplementary Figure 4.

Furthermore, we used the one-parameter rather than the Prelec-2 parameter weighting function because the former is more straightforward and simplifies the inter-subject analysis, as noted by previous studies (26, 29). Nevertheless, for validation purpose, we ran the estimation with the Prelec-2 parameter weighting function as well as a novel one-parameter weighting function with a log2 base (30). The fitting results are shown in Supplementary Table 5 and as can be seen, the model with utility function and the Prelec-2 parameter weighting function (model 8) outperformed the main model used here (model 6, AIC = 90.75 versus 96.03), while the model with utility function and the novel one-parameter weighting function with a log2 base (model 10) performed equally well with the main model (model 6, AIC = 95.96 versus 96.03). To validate our results, we therefore reran all the analysis with the parameters estimated from models 8 and 10.

For model 8 with the Prelec-2 parameter weighting function, the results of multiple linear regression using BDI/BDI-pure and STAI-Y1 to predict the risk parameters are shown in Supplementary Tables 6, 7. As can be seen, BDI and BDI-pure but not STAI-Y1 were significantly associated with γ; neither BDI/BDI-pure nor STAI-Y1 was significantly associated with λ. The results of the correlation between the PCA extracted factors and risk parameters are shown in Supplementary Table 8. Factor 3 that captures depression was significantly associated with γ but not λ, even after partialling out mother education. Lastly, Depression-selected but not Anxiety-selected was significantly associated with γ but not λ, even after partialling out mother education (Supplementary Table 4).

For model 10 with the novel one-parameter weighting function with a log2 base, the results of multiple linear regression using BDI/BDI-pure and STAI-Y1 to predict the risk parameters are shown in Supplementary Tables 9, 10. Here, neither BDI/BDI-pure nor STAI-Y1 was associated with γ or λ. The results of the correlation between the PCA extracted factors and risk parameters showed that only factors measuring depression (i.e., Factors 3, 5, and 6) were significantly associated with γ, most of which remained after partialling out mother education (Supplementary Table 11). None of the factors were associated with λ. Lastly, again, Depression-selected but not Anxiety-selected was significantly associated with γ but not λ, even after partialling out mother education (Supplementary Table 4).

In summary, across three different methods of analyzing the symptoms of depression and anxiety [i.e., (a) incorporating them simultaneously in multiple linear regression to predict the risk parameters, (b) examining the correlation between the underlying latent factors of depression and anxiety extracted using PCA and the risk parameters, and (c) selecting items with the fewest correlations with items from the other scale to create Depression-selected and Anxiety-selected and then examining the correlation between these new variables and the risk parameters] and three different probability weighting functions for computational modeling (i.e., Prelec-1 parameter, Prelec-2 parameter, and one-parameter function with a log2 base), the most consistent pattern of result observed was the association between depression and γ. Namely, as the symptoms of depression increased, subjects’ tendency to overweight small probabilities and underweight large probabilities decreased.

## Results

Among 68 participants that conducted the decision-making task, 11 failed to respond on over five trials and/or made over two incorrect choices on the eight test trials, two almost always chose the option with the higher probability which did not permit reliable model fitting, two had fitted parameters over three standard deviation (SD) above the mean of all participants which was also far greater than previously reported [e.g., Hsu et al. (26)]. These subjects were therefore excluded and the data of the remaining 53 participants were used for further analysis. Importantly, the excluded participants did not differ from the remaining participants in terms of BDI, BDI-pure, or STAI-Y1 (all *p* > 0.4). Among the remaining participants, 21 were males and 32 females, the mean age was 22.48 (SD 2.92) years. The mean scores of BDI, BDI-pure, and STAI-Y1 were 7.98 (SD 7.67), 7.57 (SD 7.02), and 37.45 (SD 8.89), respectively.

For the computational model-based analysis, as shown in Table 1, model 6 that had a utility parameter λ and probability weighting parameter γ was the winning model since it had the smallest AIC.

**FIGURE 3:**
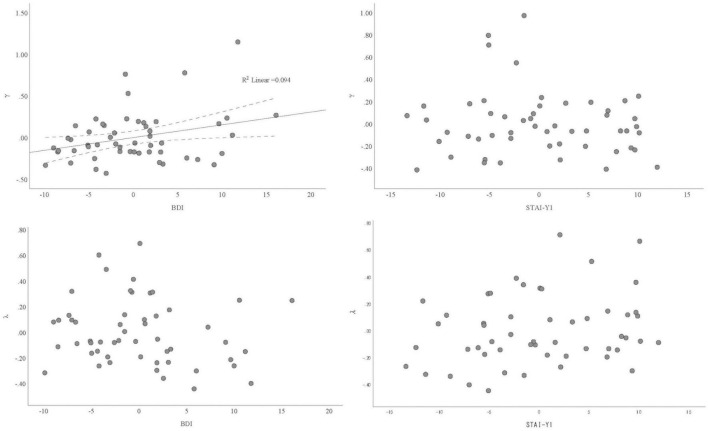
Partial regression plot of the association between depression/anxiety and γ/λ.

To investigate the independent association between depression, anxiety, λ, and γ, we conducted multiple linear regression for λ and γ, respectively, with BDI and STAI-Y1 as independent variables. As shown in Table 2 (Model 1), BDI but not STAI-Y1 was significantly associated with γ (*B* = 0.015, 95% CI = [0.002, 0.029], *p* < 0.05). Neither BDI nor STAI-Y1 was significantly associated with λ. The partial regression plot of BDI/STAI-Y1 and the parameters are shown in Figure 3. Among demographic factors, only mother education level was associated with γ, we therefore incorporated this variable as a covariate in the regression analysis. As reported in Table 2 (Model 2), even after controlling the influence of mother education, the association between BDI and γ remained significant (*B* = 0.014, 95% CI = [0.000, 0.027], *p* < 0.05). Notably, mother education was also significantly associated with γ (*B* = 0.115, 95% CI = [0.006, 0.224], *p* < 0.05), although its standardized regression coefficient was smaller than that of BDI (i.e., 0.277 versus 0.346).

Given that BDI has two items that are also considered the primary symptoms of anxiety, namely agitation and irritability, we therefore created another variable of depression, BDI-pure, by excluding these two items. We repeated the above multiple linear regression. As shown in Table 3, only BDI-pure was associated with γ (*B* = 0.017, 95% CI = [0.003, 0.032], *p* < 0.05), this remained even after controlling mother education (*B* = 0.016, 95% CI = [0.002, 0.030], *p* < 0.05).

**FIGURE 4:**
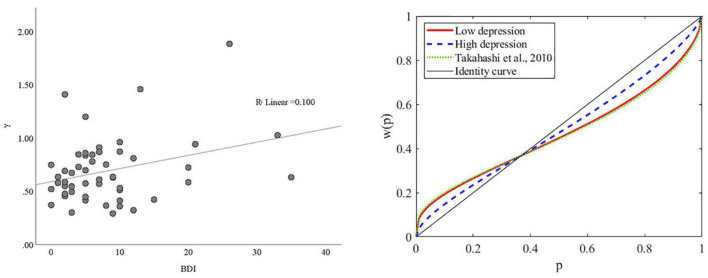
The association between BDI and γ. **(Left panel)** Scatterplot of the association between BDI and γ. **(Right panel)** Probability weighting function plots for subjects with high and low depression. The mean of γ for the low and high depression groups were 0.5964 and 0.7772, respectively. The mean of γ for a similar group of Japanese young adults was 0.57 in Takahashi et al. (29).

To provide a visual illustration of the change in γ (i.e., probability weighting) given the change in BDI, we plotted the simple scatterplot of the association between BDI and γ in Figure 4 (left panel). We also categorized participants based on their score of BDI, such that those within the lower quartile were categorized as having low depression (*n* = 17) and those within the upper quartile were categorized as having high depression (*n* = 16). The plot of the probability weighting function for each group mean is shown in Figure 4 (right panel). As can be seen, the probability weighting of the low depression group is similar to previously reported in Japanese healthy young adults (29), in which participants tend to overweight small probabilities while underweight large probabilities. In contrast, this tendency is decreased in the high depression group.

## Discussion

To our knowledge, this is the first study to assess risk preference due to a concave utility function and nonlinear probability weighting separately for depression and anxiety using computational modeling. We showed that depression, but not anxiety, is associated with the nonlinear probability weighting parameter. That is, as the symptoms of depression increases, the typical overweighting of small probabilities and underweighting of large probabilities shown in people with low depression become attenuated. The probability weighting of the low depression group is also similar to previously reported in Japanese healthy young adults (29), both consistent with the Prospect Theory (22, 23). In contrast, neither depression nor anxiety was associated with utility sensitivity.

Depression and anxiety often co-occur with each other, as a result, comorbid major depressive disorder and an anxiety disorder (13, 31) and anxious-depression [or major depressive disorder with subthreshold anxiety symptoms; (32)] are highly prevalent. This co-occurrence is associated with greater distress, higher risk of suicide, poorer treatment outcomes, and higher rate of recurrence (33–36). One psychopathological mechanism proposed to account such co-occurrence is risk-avoidant behaviors, which has been reported to be common to both depression and anxiety (see section “Introduction”). Nevertheless, the independent association between depression, anxiety, and risk preference has been seldom investigated (37). In the only study that investigated depression, anxiety, and utility sensitivity-based risk preference, Charpentier et al. (10) showed that patients with generalized anxiety disorder (GAD) had higher risk aversion (as indicated by a smaller λ) compared to healthy controls. Meanwhile, anxiety was positively associated with risk aversion when controlling depression, the opposite (i.e., the association between depression and risk aversion while controlling anxiety), however, was nonsignificant. The precise reason of such inconsistency between our and Charpentier et al.’s (10) results is unclear, future research is required to investigate if the difference in subject characteristics (healthy volunteers versus patients), task design (whether combine gain-only gambles with gain-loss mixed gambles), computational modeling (whether incorporate utility sensitivity and probability weighting simultaneously) may potentially explain the gap.

So far, in the field of computational model-based analysis of decision-making, reduced reward sensitivity and reinforcement learning rate have been considered major decision-making deficits in depression (38, 39). However, as suggested by previous studies with IGT and BART, enhanced risk aversion is likely to be another critical deficit. Here, by removing the learning component and teasing apart utility sensitivity and probability weighting, we showed that depression is associated with underweighting of small probabilities (compared to those with low symptoms of depression), or in other words, enhanced risk aversion at small probabilities. It remains for future studies to test if the assumption of reduced reward sensitivity and reinforcement learning is true or just a confounding effect of probability weighting.

Our findings are consistent with previous reports of mood influence on judgment (40–42). For instance, when in a sad mood, people tend to underestimate the probabilities of positive events, whereas in a happy mood, they tend to overestimate such probabilities (41). Several theoretical explanations have been proposed, for instance, people’s feelings are projected to the judgment (42) or their feelings are used as information for the judgment (43). From the perspective of the psychopathology of depression, the underweighting of small probabilities is a result of the depressogenic schemata or distorted cognition (44). One common depressogenic schemata is the 0-or-100 thinking (or black and white thinking, all-or-nothing mindset), that is, depressed patients tend to consider things in a dichotomous or polarized manner. With such a schemata, given two options one with high and the other low probability of getting a reward, people with high levels of depression will underestimate the low probability while overestimate the high probability when making the choice.

It is unclear why we did not observe an association between anxiety and both parameters of γ and λ. One previous study that employed the economic definition of risk has reported enhanced risk-aversion in patients with GAD and panic disorder (9). However, they did not remove the confounding effect of depressive symptoms and their sample size was relatively small (i.e., *n* = 10 for GAD and panic disorder each). Future research is required to confirm our findings and clarify the association between anxiety and risk preference.

Our study also has limitations. Firstly, we limited the age of our participants to 20–39 years to remove the confounding of age and improve statistical power, which, however, also limits the generalization of our conclusion to older adults and adolescents. Secondly, to simplify the task, we focused on decision based on only reward without considering loss. The Cumulative Prospect Theory argues that valuation differs between situations of reward and loss (23) and it is possible that our results may not apply to decisions involving loss. Thirdly, our subjects were healthy volunteers and therefore the symptoms of depression and anxiety here we evaluated were primarily in the normal range. Future studies are required to confirm if the association we observed also exist in clinical patients.

Despite these limitations, our study represents an important, first step toward the precise understanding of the cognitive computational mechanisms of decision-making in depression and anxiety. Such endeavors may help to elucidate the psychopathological basis of these disorders and facilitate the identification of their underlying neurobiological impairments.

## Data Availability Statement

The data that support the findings of this study are available from the corresponding author upon reasonable request.

## Ethics Statement

The studies involving human participants were reviewed and approved by the Institutional Review Board of Yamaguchi University Hospital. The patients/participants provided their written informed consent to participate in this study.

## Author Contributions

CC and SN: conceptualization and design. KH, CC, HL, and MH: investigation. KH, YM, and CC: data analysis and manuscript preparation. TM: resources. All authors contributed to manuscript revision.

## Conflict of Interest

The authors declare that the research was conducted in the absence of any commercial or financial relationships that could be construed as a potential conflict of interest.

## Publisher’s Note

All claims expressed in this article are solely those of the authors and do not necessarily represent those of their affiliated organizations, or those of the publisher, the editors and the reviewers. Any product that may be evaluated in this article, or claim that may be made by its manufacturer, is not guaranteed or endorsed by the publisher.

## Footnotes

1
http://psychtoolbox.org/
