# Supplementary material for: Nonlinear Probability Weighting in Depression and Anxiety: Insights From Healthy Young Adults
Source: Front Psychiatry. 2022 Mar 24;13:810867. doi: 10.3389/fpsyt.2022.810867 (PMC8988187; doi:10.3389/fpsyt.2022.810867)
Supplement: Supplementary file 1 [file Data_Sheet_1.pdf]

Nonlinear probability weighting in depression and anxiety: insights from healthy young adults

Supplementary material

Table S1. The item structures of the seven factors underlying BDI and STAI-Y1 extracted from the principle component analysis (PCA)

Table S2. Correlation between PCA extracted factors and risk parameters using the Prelec-1 parameter function

Table S3. Number of correlations of individual items of BDI and STAI-Y1 with items from the other scale, respectively

Table S4. Correlation between Depressed-selected/Anxiety-selected and risk parameters

Table S5. Additional computational models fitted

Table S6. Results of the multiple linear regression using BDI with risk parameters using the Prelec-2 parameter function

Table S7. Results of the multiple linear regression using BDI-pure with risk parameters using the Prelec-2 parameter function

Table S8. Correlation between PCA extracted factors and risk parameters using the Prelec-2 parameter function

Table S9. Results of the multiple linear regression using BDI with risk parameters using the one-parameter probability weighting function having a log2 base

Table S10. Results of the multiple linear regression using BDI-pure with risk parameters using the one-parameter probability weighting function having a log2 base

Table S11. Correlation between PCA extracted factors and risk parameters using the one-parameter probability weighting function having a log2 base

Figure S1. Normal P-P plot of regression standardized residual of multiple linear regressions shown in Table 2.

Figure S2. Normal P-P plot of regression standardized residual of multiple linear regressions shown in Table 3.

Figure S3. The scree plot of the principle component analysis (PCA).

Figure S4. Scatterplot of the association between Depressed-selected/Anxiety-selected and risk parameters using the Prelec-1 parameter function

Table S1. The item structures of the seven factors underlying BDI and STAI-Y1extracted from the principle component analysis (PCA)

Domain	Anxiety	Anxiety/Depression	Depression	Depression	Depression	Depression	Anxiety
Factors	Factor 1	Factor 2	Factor 3	Factor 4	Factor 5	Factor 6	Factor 7
Items	STAI-Y1 1 Calm<br>STAI-Y1 2 Secure<br>STAI-Y1 5 At ease<br>STAI-Y1 8 Satisfied<br>STAI-Y1 10 Comfortable<br>STAI-Y1 11 Self-confident<br>STAI-Y1 15 Relaxed<br>STAI-Y1 16 Content<br>STAI-Y1 17 Worried<br>STAI-Y1 19 Steady<br>STAI-Y1 20 Pleasant	STAI-Y1 7 Worrying<br>STAI-Y1 9 Frightened<br>STAI-Y1 12 Nervous<br>STAI-Y1 13 Jittery<br>STAI-Y1 14 Indecisive<br>BDI 6 Punishment feelings<br>BDI 10 Crying<br>BDI 15 Loss of energy<br>BDI 17 Irritability	STAI-Y1 6 Upset<br>BDI 1 Sadness<br>BDI 7 Self-dislike<br>BDI 21 Loss of interest in sex	BDI 3 Past failure<br>BDI 5 Guilty<br>BDI 14 Worthlessness	STAI-Y1 4 Strained<br>BDI 2 Pessimism<br>BDI 16 Sleep	BDI 4 Loss of pleasure<br>BDI 9 Suicidal thoughts	STAI-Y1 3 Tense<br>BDI 11 Agitation<br>BDI 13 Indecisiveness

Items of depression and anxiety are shown in blue and red, respectively.

Table S2. Correlation between PCA extracted factors and risk parameters using the Prelec-1 parameter function

Domain	Anxiety	Anxiety/Depression	Depression	Depression	Depression	Depression	Anxiety
Factors	Factor 1	Factor 2	Factor 3	Factor 4	Factor 5	Factor 6	Factor 7
Sum of raw scores of items loading on the factor							
γ	r=0.094, p=0.505	r=0.092, p=0.511	**r=0.275, p=046***	r=0.106, p=0.448	**r=0.274, p=0.047***	r=0.257, p=0.063	r=0.114, p=0.416
λ	r=0.205, p=0.141	r=-0.170, p=0.225	r=-0.112, p=0.424	r=0.016, p=0.911	r=0.137, p=0.328	r=-0.222, p=0.110	r=-0.164, p=0.241
γ (partialling out mother education)	r=0.133, p=0.348	r=0.108, p=0.445	r=0.250, p=0.074	r=0.150, p=0.288	r=0.260, p=0.063	r=0.252, p=0.072	r=0.079, p=0.580
λ (partialling out mother education)	r=0.194, p=0.169	r=-0.179, p=0.204	r=-0.107, p=0.451	r=-0.005, p=0.974	r=0.149, p=0.291	r=-0.215, p=0.126	r=-0.154, p=0.276
Anderson-Rubin score							
γ	r=0.086, p=0.542	r=-0.210, p=0.132	**r=0.278, p=0.044** *	r=-0.047, p=0.736	r=0.249, p=0.072	r=0.249, p=0.072	r=0.110, p=0.433
λ	r=0.251, p=0.070	r=0.013, p=0.929	r=-0.144, p=0.302	r=-0.071, p=0.615	r=0.194, p=0.164	r=0.044, p=0.756	r=-0.109, p=0.437
γ (partialling out mother education)	r=0.114, p=0.420	r=-0.232, p=0.098	r=0.247, p=0.078	r=-0.016, p=0.912	r=0.215, p=0.126	r=0.228, p=0.104	r=0.119, p=0.399
λ (partialling out mother education)	r=0.242, p=0.084	r=0.018, p=0.902	r=-0.129, p=0.361	r=-0.088, p=0.533	r=0.217, p=0.122	r=0.060, p=0.673	r=-0.114, p=0.422

* $p<0.05$. Coefficients are based on Spearman's rank order correlation due to non-normal distribution of the data.

Table S3. Number of correlations of individual items of BDI and STAI-Y1 with items from the other scale, respectively

BDI items	Number of correlated items of STAI-Y1	BDI items	Number of correlated items of STAI-Y1	STAI-Y1 items	Number of correlated items of BDI	STAI-Y1 items	Number of correlated items of BDI
16 Sleep	1	7 Self-dislike	6	2 Secure	0	12 Nervous	7
20 Fatigue	1	3 Past failure	6	18 Confused	0	11 Self-confident	9
18 Appetite	3	2 Pessimism	7	1 Calm	1	13 Jittery	9
21 Loss of interest in sex	3	8 Self-criticalness	8	3 Tense	1	16 Content	9
1 Sadness	4	15 Loss of energy	8	4 Strained	2	10 Comfortable	9
9 Suicidal thoughts	4	11 Agitation	8	5 At east	2	17 Worried	11
12 Loss of interest	4	4 Loss of pleasure	9	8 Satisfied	4	9 Frightened	11
14 Worthlessness	4	19 Concentration	10	20 Pleasant	4	6 Upset	12
13 Indecisiveness	5	17 Irritability	12	15 Relaxed	5	14 Indecisiveness	12
10 Crying	6	5 Guilty	15	19 Steady	6	7 Worrying	16
6 Punishment feelings	6						

Selected items of depression and anxiety are shown in blue and red, respectively.

Table S4. Correlation between Depressed-selected/Anxiety-selected and risk parameters

Factors	Depressed-selected	Anxiety-selected
Prelec-1 parameter function		
γ	**r=0.329, p=0.016***	r=0.039, p=0.780
λ	r=-0.074, p=0.597	r=0.105, p=0.456
γ (partialling out mother education)	**r=0.331, p=0.017***	r=0.058, p=0.682
λ (partialling out mother education)	r=-0.074, p=0.603	r=0.098, p=0.488
Prelec-2 parameter function		
γ	**r=0.290, p=0.035***	r=-0.061, p=0.666
a	r=-0.239, p=0.085	r=-0.119, p=0.395
λ	r=-0.229, p=0.099	r=0.022, p=0.875
γ (partialling out mother education)	**r=0.297, p=0.033***	r=-0.061, p=0.668
a (partialling out mother education)	r=-0.241, p=0.085	r=-0.131, p=0.353
λ (partialling out mother education)	r=-0.228, p=0.104	r=0.018, p=0.895
One-parameter function with a log2 base		
γ	**r=0.282, p=0.041***	r=0.121, p=0.389
λ	r=-0.065, p=0.645	r=0.070, p=0.620
γ (partialling out mother education)	**r=0.293, p=0.035***	r=0.137, p=0.334
λ (partialling out mother education)	r=-0.066, p=0.643	r=0.066, p=0.641

* $p<0.05$. Coefficients are based on Spearman’s rank order correlation due to non-normal distribution of the data.

Table S5. Additional computational models fitted

Model No.	Model description	Equation	Free parameters	AIC
7	Magnitude and Probability with probability weighting (Prelec-2)	$V(X) = re^{-a(-\log p)^{\gamma}}$	a,γ,β	102.33
**8**	**Magnitude with utility function and Probability with probability weighting (Prelec-2)**	$\boldsymbol{V(X) = r^{\lambda}e^{-a(-\log p)^{\gamma}}}$	**λ,a,γ,β**	**90.75**
9	Magnitude and Probability with probability weighting (novel 1-parameter)	$V(X) = r2^{-((-log_2(p))^{\gamma})}$	γ,β	148.31
**10**	**Magnitude with utility function and Probability with probability weighting (novel 1-parameter)**	$\boldsymbol{V(X) = r^{\lambda}2^{-((-log_2(p)^{\gamma})}}$	**λ,γ,β**	**95.96**

Table S6. Results of the multiple linear regression using BDI with risk parameters using the Prelec-2 parameter function

	Independent variables	Dependent variable: γ			Dependent variable: a			Dependent variable: λ		
		Unstandardized B (95% CI)	Standardized beta	p	Unstandardized B (95% CI)	Standardized beta	p	Unstandardized B (95% CI)	Standardized beta	p
Model 1	BDI	**0.033 (0.007, 0.059)**	**0.429**	**0.015***	-0.003 (-0.098, 0.093)	-0.010	0.956	-0.007 (-0.045, 0.030)	-0.071	0.692
	STAI-Y1	-0.012 (-0.034, 0.011)	-0.177	0.302	-0.033 (-0.116, 0.050)	-0.144	0.425	-0.007 (-0.039, 0.025)	-0.077	0.671
Model 2	BDI	**0.032 (0.006, 0.058)**	**0.419**	**0.019***	0.000 (-0.097, 0.098)	0.001	0.996	-0.008 (-0.046,0.030)	-0.077	0.675
	STAI-Y1	-0.011 (-0.034, 0.012)	-0.166	0.340	-0.036 (-0.120, 0.048)	-0.156	0.396	-0.006 (-0.039,0.026)	-0.070	0.111
	Mother education	0.051 (-0.168, 0.270)	0.063	0.642	-0.196 (-1.003, 0.610)	-0.070	0.627	0.040 (-0.274,0.354)	0.036	0.800

* p<0.05

Table S7. Results of the multiple linear regression using BDI-pure with risk parameters using the Prelec-2 parameter function

	Independent variables	Dependent variable: γ			Dependent variable: a			Dependent variable: λ		
		Unstandardized B (95% CI)	Standardized beta	p	Unstandardized B (95% CI)	Standardized beta	p	Unstandardized B (95% CI)	Standardized beta	p
Model 1	BDI-pure	**0.037 (0.010, 0.065)**	**0.447**	**0.009****	-0.003 (-0.106, 0.099)	-0.011	0.949	-0.008 (-0.048, 0.032)	-0.071	0.688
	STAI-Y1	-0.012 (-0.034, 0.010)	-0.179	0.283	-0.033 (-0.114, 0.048)	-0.143	0.417	-0.007 (-0.038, 0.024)	-0.078	0.657
Model 2	BDI-pure	**0.036 (0.008, 0.065)**	**0.438**	**0.012***	0.000 (-0.104, 0.104)	0.000	1.000	-0.009 (-0.049,0.032)	-0.077	0.669
	STAI-Y1	-0.011 (-0.033, 0.011)	-0.169	0.319	-0.036 (-0.118, 0.047)	-0.155	0.388	-0.006 (-0.039,0.026)	-0.072	0.689
	Mother education	0.047 (-0.170, 0.264)	0.058	0.665	-0.196 (-1.003, 0.611)	-0.070	0.628	0.040 (-0.274,0.355)	0.037	0.798

* $p<0.05$, ** $p<0.01$

Table S8. Correlation between PCA extracted factors and risk parameters using the Prelec-2 parameter function

Domain	Anxiety	Anxiety/Depression	Depression	Depression	Depression	Depression	Anxiety
Factors	Factor 1	Factor 2	Factor 3	Factor 4	Factor 5	Factor 6	Factor 7
Sum of raw scores of items loading on the factor							
γ	r=-0.015, p=0.915	r=0.137, p=0.329	**r=0.378, p=0.005****	r=0.154, p=0.272	r=0.220, p=0.114	r=0.170, p=0.225	r=-0.080, p=0.570
a	r=-0.170, p=0.223	r=-0.029, p=0.834	r=-0.082, p=0.559	r=-0.097, p=0.488	r=-0.244, p=0.078	r=-0.269, p=0.051	r=-0.218, p=0.117
λ	r=0.029, p=0.838	r=0.003, p=0.982	r=-0.165, p=0.237	r=-0.037, p=0.792	r=-0.129, p=0.356	r=-0.231, p=0.096	r=-0.114, p=0.418
γ (partialling out mother education)	r=-0.008, p=0.953	r=0.145, p=0.305	**r=0.381, p=0.005****	r=0.169, p=0.232	r=0.219, p=0.119	r=0.167, p=0.237	r=-0.090, p=0.525
a (partialling out mother education)	r=-0.200, p=0.154	r=-0.035, p=0.807	r=-0.059, p=0.677	r=-0.130, p=0.359	r=-0.237, p=0.091	r=-0.263, p=0.060	r=-0.190, p=0.178
λ (partialling out mother education)	r=0.019, p=0.896	r=0.002, p=0.988	r=-0.155, p=0.272	r=-0.052, p=0.714	r=-0.121, p=0.391	r=-0.228, p=0.105	r=-0.100, p=0.481
Anderson-Rubin score							
γ	r=-0.032, p=0.820	r=-0.075, p=0.592	**r=0.305, p=0.026***	r=0.050, p=0.721	r=0.207, p=0.137	r=0.173, p=0.215	r=-0.016, p=0.910
a	r=-0.122, p=0.384	**r=0.290, p=0.035***	r=-0.027, p=0.847	r=0.010, p=0.945	r=-0.093, p=0.510	r=-0.264, p=0.056	r=-0.223, p=0.108
λ	r=0.082, p=0.557	**r=0.300, p=0.029***	r=-0.209, p=0.134	r=-0.057, p=0.685	r=0.024, p=0.863	r=-0.143, p=0.807	r=-0.166, p=0.234
γ (partialling out mother education)	r=-0.029, p=0.840	r=-0.077, p=0.587	**r=0.303, p=0.029***	r=0.064, p=0.653	r=0.204, p=0.147	r=0.162, p=0.251	r=-0.018, p=0.900

a (partialling out mother education)	r=-0.143, p=0.312	**r=0.306, p=0.027***	r=0.005, p=0.997	r=-0.017, p=0.905	r=-0.063, p=0.659	r=-0.249, p=0.075	r=-0.223, p=0.113
λ (partialling out mother education)	r=0.077, p=0.590	**r=0.308, p=0.026***	r=-0.200, p=0.155	r=-0.073, p=0.607	r=0.041, p=0.771	r=-0.135, p=0.340	r=-0.168, p=0.234

* p<0.05, ** p<0.01. Coefficients are based on Spearman's rank order correlation due to non-normal distribution of the data.

Table S9. Results of the multiple linear regression using BDI with risk parameters using the one-parameter probability weighting function having a log2 base

	Independent variables	Dependent variable: γ			Dependent variable: λ		
		Unstandardized B (95% CI)	Standardized beta	p	Unstandardized B (95% CI)	Standardized beta	p
Model 1	BDI	0.009 (-0.008, 0.027)	0.192	0.276	-0.006 (-0.018, 0.006)	-0.184	0.305
	STAI-Y1	0.004 (-0.011, 0.019)	0.101	0.564	0.007 (-0.003, 0.017)	0.237	0.190
Model 2	BDI	0.008 (-0.009, 0.025)	0.159	0.364	-0.006 (-0.018,0.006)	-0.185	0.311
	STAI-Y1	0.006 (-0.009, 0.021)	0.138	0.431	0.007 (-0.004,0.017)	0.237	0.196
	Mother education	0.111 (-0.031, 0.252)	0.214	0.122	0.002 (-0.099,0.103)	0.005	0.971

Table S10. Results of the multiple linear regression using BDI-pure with risk parameters using the one-parameter probability weighting function having a log2 base

	Independent variables	Dependent variable: γ			Dependent variable: λ		
		Unstandardized B (95% CI)	Standardized beta	p	Unstandardized B (95% CI)	Standardized beta	p
Model 1	BDI-pure	0.010 (-0.009, 0.028)	0.181	0.295	-0.006 (-0.019, 0.007)	-0.156	0.377
	STAI-Y1	0.005 (-0.010, 0.019)	0.112	0.514	0.006 (-0.004, 0.016)	0.215	0.224
Model 2	BDI-pure	0.008 (-0.010, 0.026)	0.147	0.392	-0.006 (-0.019,0.007)	-0.156	0.384
	STAI-Y1	0.006(-0.008, 0.021)	0.149	0.387	0.006 (-0.004,0.017)	0.216	0.231
	Mother education	0.111 (-0.031, 0.252)	0.214	0.122	0.001 (-0.100,0.103)	0.003	0.981

Table S11. Correlation between PCA extracted factors and risk parameters using the one-parameter probability weighting function having a log2 base

Domain	Anxiety	Anxiety/Depression	Depression	Depression	Depression	Depression	Anxiety
Factors	Factor 1	Factor 2	Factor 3	Factor 4	Factor 5	Factor 6	Factor 7
Sum of raw scores of items loading on the factor							
γ	r=0.115, p=0.412	r=0.159, p=0.256	**r=0.277, p=0.044***	r=0.089, p=0.525	**r=0.308, p=0.025***	**r=0.282, p=0.041***	r=0.156, p=0.266
λ	r=0.185, p=0.185	r=-0.210, p=0.131	r=-0.152, p=0.277	r=-0.016, p=0.907	r=0.140, p=0.317	r=-0.220, p=0.113	r=-0.171, p=0.220
γ (partialling out mother education)	r=0.158, p=0.265	r=0.173, p=0.221	r=0.255, p=0.068	r=0.137, p=0.334	**r=0.306, p=0.028***	**r=0.281, p=0.044***	r=0.110, p=0.437
λ (partialling out mother education)	r=0.182, p=0.198	r=-0.221, p=0.116	r=-0.151, p=0.285	r=-0.026, p=0.855	r=0.142, p=0.315	r=-0.220, p=0.117	r=-0.162, p=0.251
Anderson-Rubin score							
γ	r=0.125, p=0.372	r=-0.101, p=0.472	**r=0.306, p=0.026***	r=-0.108, p=0.440	r=0.184, p=0.187	r=0.249, p=0.073	r=0.151, p=0.281
λ	r=0.225, p=0.105	r=-0.050, p=0.720	r=-0.181, p=0.194	r=-0.088, p=0.531	r=0.245, p=0.077	r=0.041, p=0.769	r=-0.135, p=0.335
γ (partialling out mother education)	r=0.156, p=0.271	r=-0.117, p=0.408	**r=0.281, p=0.044***	r=-0.074, p=0.602	r=0.150, p=0.288	r=0.234, p=0.095	r=0.147, p=0.300
λ (partialling out mother education)	r=0.224, p=0.111	r=-0.052, p=0.714	r=-0.176, p=0.211	r=-0.097, p=0.494	r=0.262, p=0.061	r=0.049, p=0.732	r=-0.133, p=0.348

* $p<0.05$. Coefficients are based on Spearman's rank order correlation due to non-normal distribution of the data.

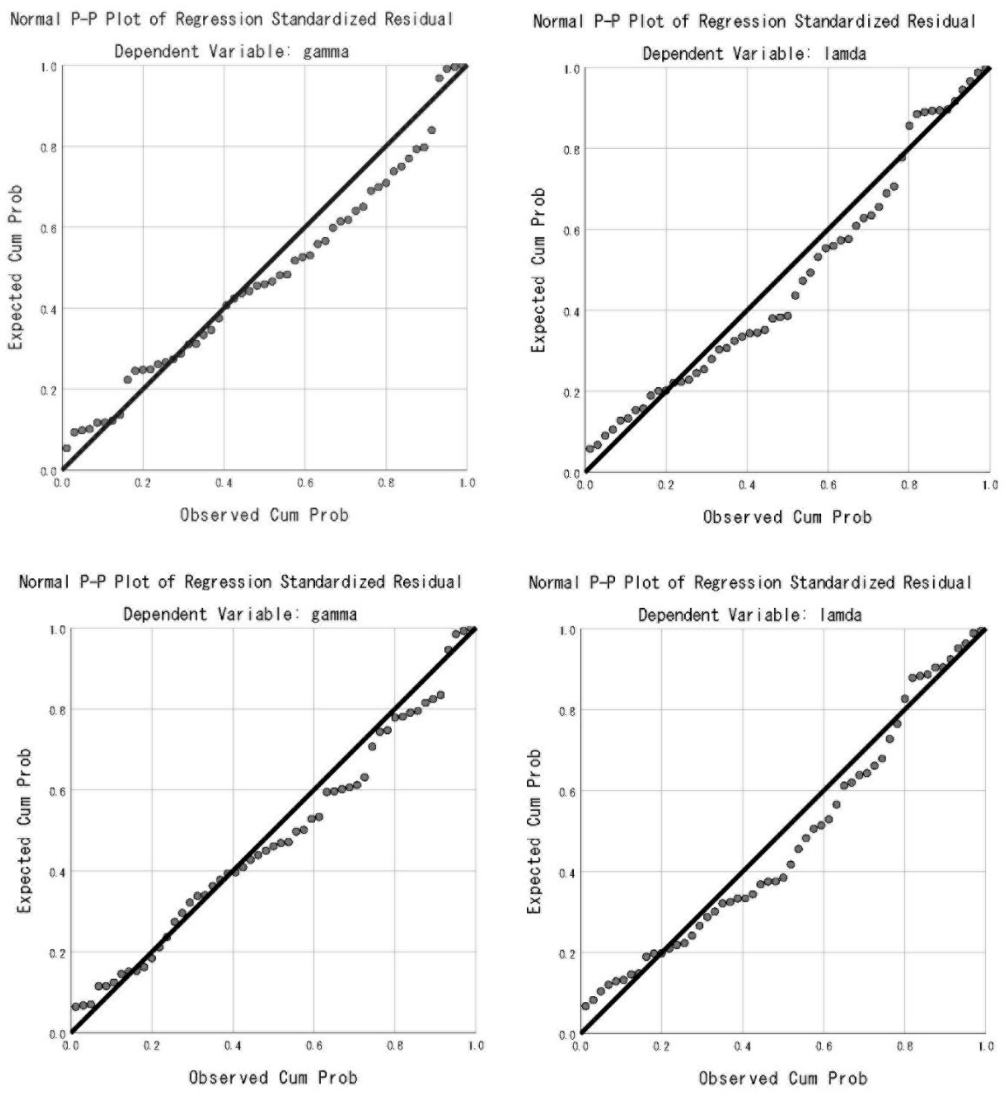

Figure S1. Normal P-P plot of regression standardized residual of multiple linear regressions shown in Table 2.

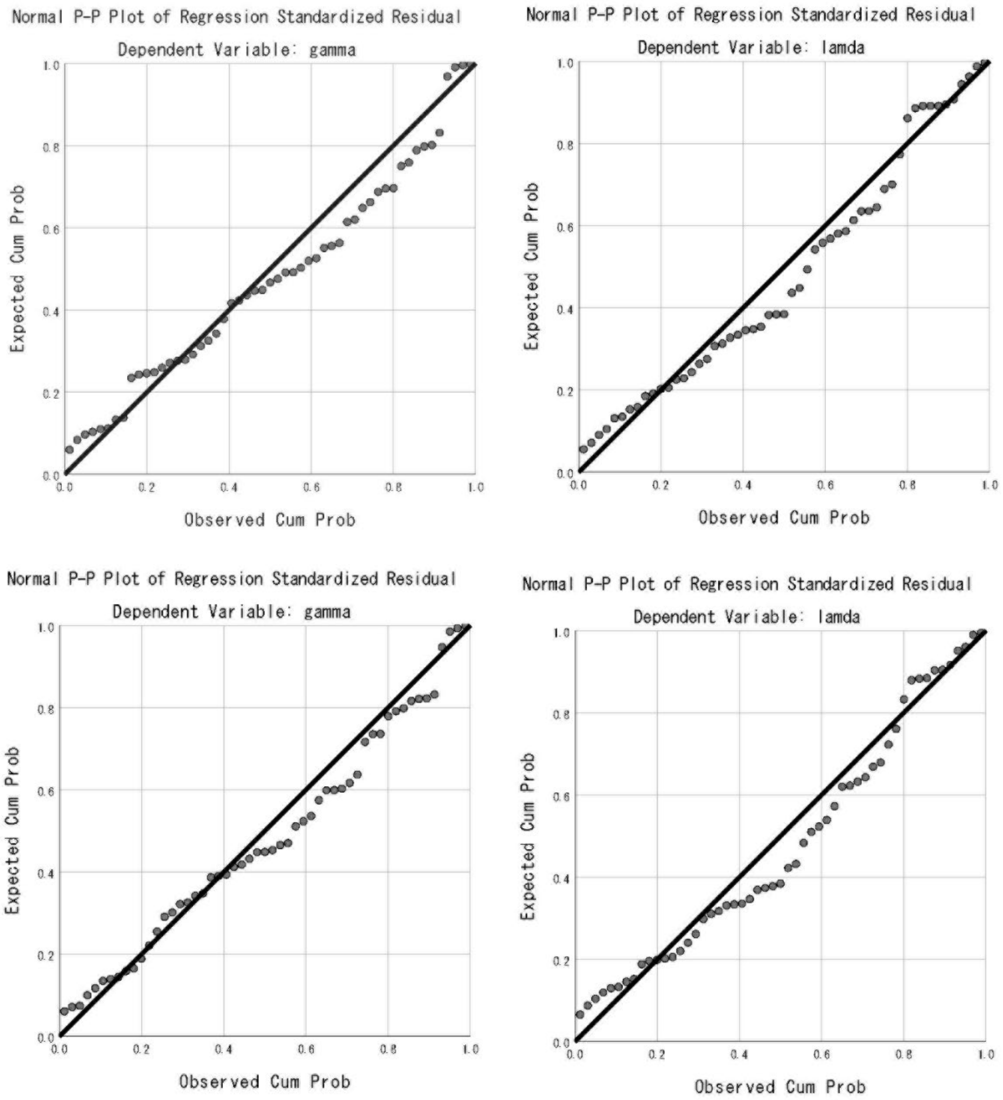

Figure S2. Normal P-P plot of regression standardized residual of multiple linear regressions shown in Table 3.

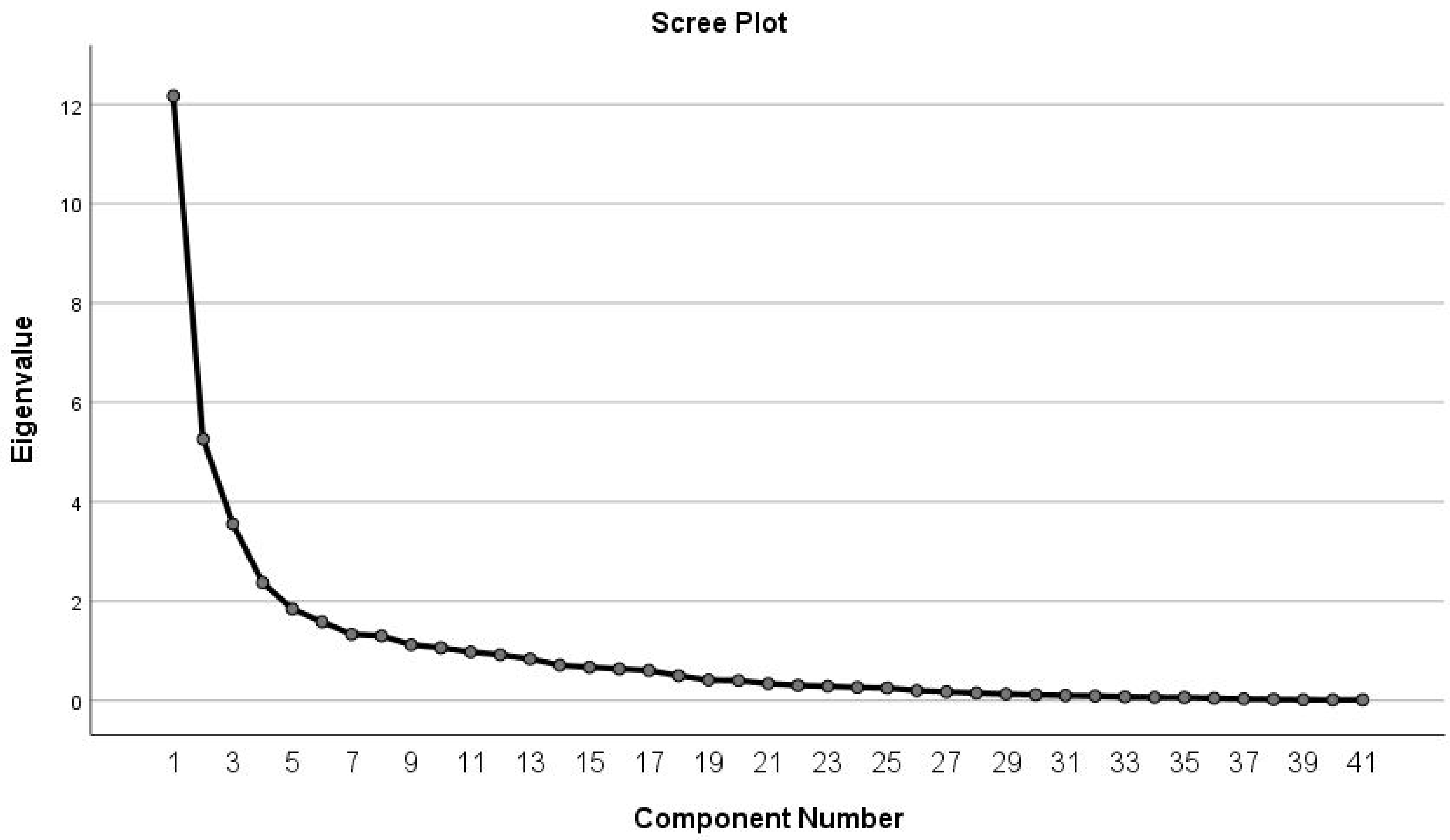

Figure S3. The scree plot of the principle component analysis (PCA). Seven factors were extracted, accounting for 64% of the total variance.

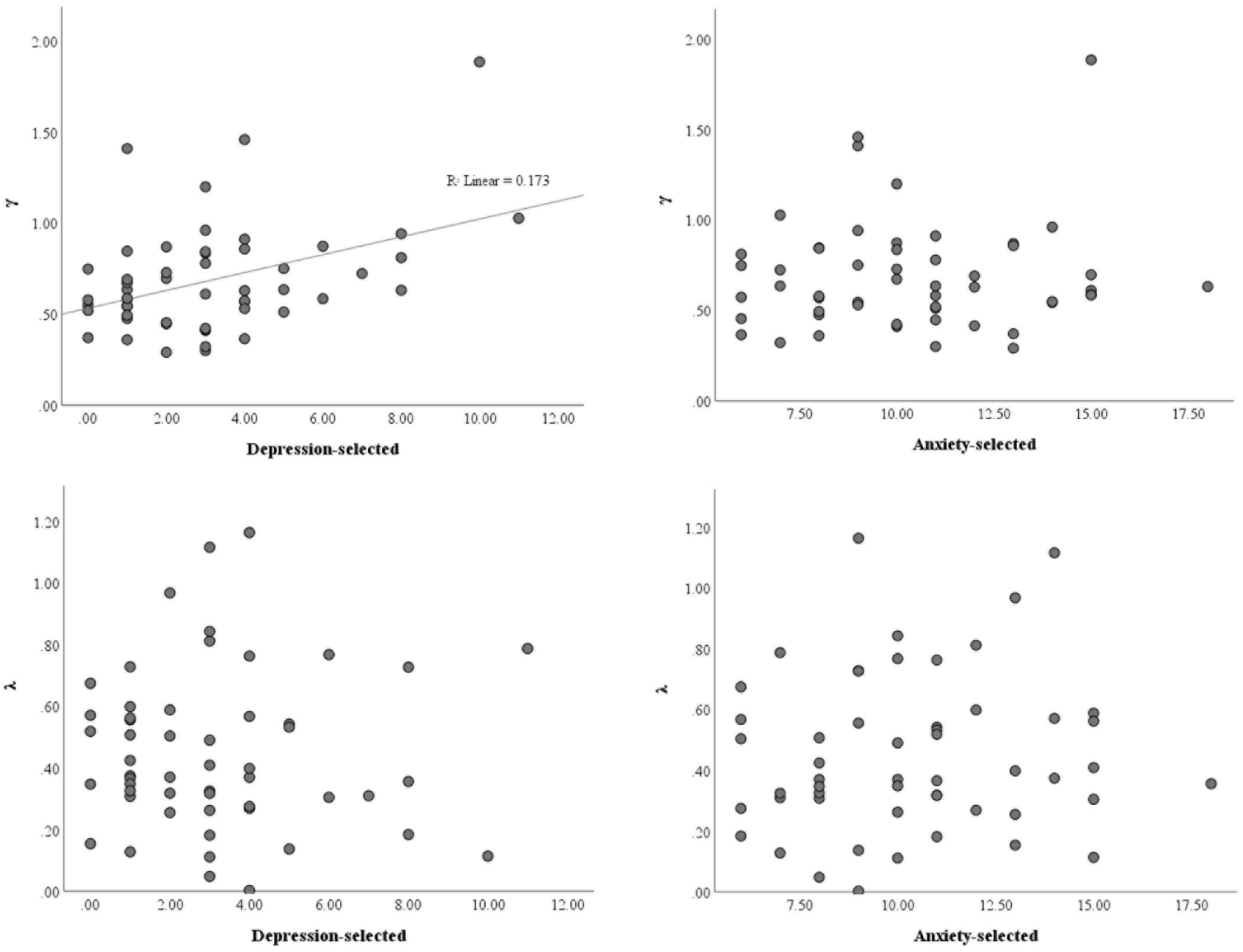

Figure S4. Scatterplot of the association between Depressed-selected/Anxiety-selected and risk parameters using the Prelec-1 parameter function
